# Public Health Impact of Wildfire Smoke Exposure: Analysis of Respiratory-Related Medicaid Claims in Wyoming

**DOI:** 10.7759/cureus.102912

**Published:** 2026-02-03

**Authors:** Joseph E Mayes, Gudeta D Fufaa

**Affiliations:** 1 Public Health, American Public University, American Military University, Ranson, USA

**Keywords:** environmental exposures, lagged effect, medicaid claims, public health, respiratory morbidity, structural barriers, wildfire smoke exposure

## Abstract

Research question: Does the rate of respiratory claims in the seven days immediately following a wildfire smoke episode significantly exceed the claims rate during the acute exposure period? The hypothesis posits that structural barriers inherent to frontier healthcare suppress immediate claims demand, resulting in a predictable lagged effect.

Methods: Daily respiratory claims data were analyzed across 23 Wyoming counties during the 2012-2024 wildfire seasons. The primary analytical approach employed a negative binomial generalized mixed-effects model with county-level fixed effects to compare the incidence rate of claims during the smoke episode (the exposure period) with that during the subsequent seven-day post-episode period.

Results: The analysis revealed a statistically significant five-fold increase in respiratory claims (p < 0.001), with predicted mean daily claims rising from approximately 1.6 during smoke episodes to 7.9 in the subsequent seven-day period, with an incidence rate ratio of 5.02. Specifically, the claims rate in the seven days post-episode was higher in the negative binomial model than the rate observed during the actual exposure period. This finding confirms the existence of a highly predictable, delayed public health phenomenon.

Conclusion: The observed surge in claims is interpreted as the release of suppressed demand, driven by the intersection of particulate matter 2.5’s biological latency and the overwhelming structural barriers (e.g., urgent access, lack of transportation) faced by Medicaid recipients. This research supports consideration of a paradigm shift from acute crisis response to a lag-adjusted preparedness model, requiring public health systems to implement targeted post-occurrence outreach and proactive structural interventions to mitigate this preventable surge in post-event healthcare utilization.

## Introduction

Driven by climate change and evolving land management practices, the increasing frequency and severity of wildfires have made widespread exposure to fine particulate matter (PM_2.5_​) a significant global public health concern. These particles are small enough to evade the body’s natural defenses and enter the bloodstream, frequently aggravating severe respiratory and cardiovascular disorders. While the Environmental Protection Agency (EPA) utilizes the Air Quality Index (AQI) - established under the Clean Air Act - to communicate immediate hazards via color-coded health categories, this framework primarily emphasizes acute, short-term risks. Consequently, further research is required to understand the delayed or lagged health consequences of smoke exposure, a vulnerability particularly relevant in frontier states like Wyoming, where structural barriers to healthcare may postpone medical intervention until days after the initial event.

In 2024, Wyoming reported 68,773 individuals enrolled in Medicaid and Kid Care Children's Health Insurance Program (CHIP), which is approximately 11.7% of the total population [1]. These are combined federal-state initiatives designed to provide qualified low-income individuals and families with access to reasonably priced health insurance. Medicaid assists low-income individuals and families with the cost of medical treatment, including children, pregnant women, the elderly, people who are blind, and those with disabilities. With limited exclusions for emergency care for non-citizens, eligibility is based on factors such as family income, Wyoming residency, and U.S. citizenship. Kid Care CHIP provides health insurance to children under 19 whose families earn too much to qualify for Medicaid but need affordable coverage. Children must reside in Wyoming, be citizens or permanent residents of the United States, and, with a few exceptions, not have any other health insurance to be eligible. Both programs include vulnerable population groups that may be at increased risk of adverse effects from wildfire smoke and respiratory distress or illness.

In rural and frontier states like Wyoming, the healthcare system's inherent structural restrictions make the Medicaid population even more vulnerable in the event of an environmental disaster. Residents in these areas must travel long distances for regular or urgent medical treatment due to low physician-to-patient ratios and a lack of specialized pulmonary care. This geographic remoteness turns a physiological stressor into a significant care barrier during an extended wildfire smoke incident. High PM_2.5_ levels may ground air ambulances or discourage vulnerable individuals from traveling long distances to distant medical facilities, especially if they lack access to dependable transportation or have rigid work schedules. This dynamic implies that the physiological effects of smoke exposure may be delayed and treated as a postponed medical emergency due to structural differences in healthcare access, rendering this group more vulnerable to the cascading public health effects of climate change.

This study addresses a critical gap in existing environmental health literature. While prior research has established strong evidence of respiratory morbidity during active smoke periods, it has focused primarily on acute urban hospitalizations and emergency room visits. These studies have consistently missed the "invisible" public health burden in frontier populations, where structural barriers - rather than biological latency alone - dictate the timing of healthcare utilization. By centering on a "Suppression and Release Cycle" theory, this research posits that claims demand is suppressed during environmental threats and is only "released" as a concentrated surge once the immediate barriers, such as poor visibility and grounded air transport, recede. By quantifying the incidence of respiratory claims in the seven-day "post-episode" phase, this study explicitly identifies the delayed medical emergencies that prior acute-focused surveillance has overlooked.

Purpose of the study

This study aims to rigorously examine and quantify the temporal connection between respiratory healthcare utilization and PM_2.5_ exposure associated with wildfires during the wildfire season in Wyoming among Medicaid-eligible individuals. Specifically, the core purpose is two-fold: to close a critical gap in the environmental health literature and to provide an evidence-based mandate for policy reform in frontier states.

Closing the research gap: the lagged effect and structural barriers

There is ample evidence of the immediate health consequences of wildfire smoke, but significantly less is known about the delayed effects that manifest in the days following the cessation of acute exposure. This research hypothesizes that, for the rural, low-income Medicaid population, a combination of structural barriers (including vast geographic distances, poor healthcare provider-to-patient ratios, limited non-emergency transportation, and economic constraints on missing work) effectively suppresses claims demand during the smoke event. By employing a mixed-effects statistical model to analyze Medicaid claims data in conjunction with discrete wildfire episodes, with a specific emphasis on the seven-day post-exposure period, this study aims to quantify the magnitude of this claim’s suppression and subsequent release, and identifies the anticipated post-event surge as a key finding. The use of county-level fixed effects will ensure that the identified temporal impact is robust and not merely a result of baseline differences in county size or preexisting respiratory disease prevalence.

Informing public health policy and resource allocation

The ultimate goal of this research is to enable the development of more focused and temporally accurate public health surveillance and intervention strategies. The study seeks to shift the current policy paradigm from a 'during-crisis' response to a 'lag-adjusted' preparedness model. By definitively quantifying the incidence rate ratio (IRR) of respiratory claims in the post-episode period, this research will provide the necessary evidence to mandate: (1) healthcare system preparation (ensuring adequate staffing and drug supply in the week after the smoke clears), (2) the implementation of targeted post-occurrence outreach (messaging focused on delayed symptoms), and (3) the development of proactive structural interventions (such as subsidized high-efficiency particulate air (HEPA) filtration or clean air hubs) to address the root causes of vulnerability in groups disproportionately impacted by environmental risks.

Research hypothesis

The null hypothesis (H_0_) states that after controlling for county-level fixed effects, seasonality, episode duration, and the average PM_2.5_ AQI, there is no statistically significant difference in the incidence rate of respiratory-related Medicaid claims between the wildfire smoke exposure period and the subsequent seven-day post-episode period.

The alternative hypothesis (H_1_) states that, after controlling for county-level fixed effects, seasonality, episode duration, and the average PM_2.5_ AQI, the incidence rate of respiratory-related Medicaid claims in the seven days following a wildfire smoke episode is significantly different from (specifically, predicted to be higher than) the rate observed during the initial smoke exposure period.

Literature review

There is an urgency to investigate the public health consequences of wildfires, especially regarding air pollution and respiratory effects, as the number and extent of these wildfires are rising across the United States. PM_2.5_, abundant in wildfire smoke, poses acute and long-term health hazards with uneven distribution across people and geographic areas. This review incorporates existing research from five interconnected regions to guide the study's present scope and direction: (1) the direct and indirect respiratory health effects of exposure to wildfire smoke; (2) the temporal patterns and lagged effects that impact exposure-response relationships; (3) the increased susceptibility of particular populations, influenced by structural and socioeconomic disparities; (4) the role of public health surveillance systems in identifying and mitigating health risks related to wildfires; and (5) the use of Medicaid claims data to evaluate respiratory outcomes at the population level.

Wildfire Impacts on Health

Globally, the frequency, intensity, and geographic distribution of wildfires are rising due to a combination of land-use changes, population increase, and climate change. Due to their extensive and complex health effects, these occurrences are increasingly acknowledged as serious public health risks [2]. By lengthening fire seasons, increasing the aridity of fuels, and altering precipitation patterns, climate change has intensified fire conditions. For instance, human-caused warming has shifted fire seasons in California by as much as 46 days earlier than in the 1990s [3]. Even in areas that have historically been fire-resistant, droughts, decreased snowpack, and increased vapor pressure deficits have made many landscapes more susceptible to combustion [4].

Wildfire danger has increased due to population growth, particularly as it has expanded into the wildland-urban interface (WUI), the region where undeveloped wildlands and human development intersect. In the United States, residential construction and land area in high-hazard WUI regions grew by 74% between 1990 and 2010, increasing human contact and sources of ignition [5]. Rebuilding projects often increase risk by constructing new buildings in existing fire-prone areas, leading to higher rates of fire incidents and greater infrastructure vulnerability due to this population transition [6]. Forest clearing, agricultural expansion, and urban development are examples of land-use shifts that have altered natural fire patterns and increased fuel loads. In addition to previous fire control practices that have led to artificial fuel buildup, invasive species further increase flammability, rendering forests more vulnerable to high-impact, treetop-to-treetop fires [7].

The intricate chemical composition of wildfire smoke is the primary factor influencing its adverse health effects. The bulk of the particle mass in wildfire smoke consists of PM_2.5_, which is particularly hazardous since it can circulate in the bloodstream and travel deep into the respiratory tract, causing oxidative stress and widespread inflammation [8]. Due to its high organic carbon content and reactive chemicals, wildfire PM_2.5_ is often more harmful than urban PM_2.5_, further exacerbating respiratory and cardiovascular strain. Wildfire smoke also contains O_3_, NO_2_, SO_2_, and CO, in addition to PM_2.5_.

Several research gaps remain in the effects of wildfires. First, there is a lack of research on the temporal dynamics of delayed respiratory impacts, particularly in the days following wildfire occurrences, as most studies focus on acute exposure and rapid health consequences. Understanding how chronic illnesses may worsen or manifest once the smoke has cleared is particularly important. Second, there is a lack of temporal sensitivity in the integration between wildfire exposure measurements and healthcare utilization data, such as Medicaid claims. Numerous studies focus on emergency room visits or hospitalizations, which may miss less obvious trends in outpatient care or the delayed onset of symptoms. Third, research on wildfire health underrepresents those who are vulnerable, such as Medicaid beneficiaries and low-income communities. This lack of research extends to frontier populations, such as those in Wyoming. In the context of this study, the known physiological severity of PM_2.5​_ suggests that while medical needs are immediate, they may not be immediately recorded in healthcare data. This discrepancy forms the basis of the "Suppression" phase, in which the severity of the environmental event temporarily masks the biological urgency of the smoke's impact.

Temporal Relationships and Lagged Effects

It is increasingly recognized that there is a complex, nonlinear relationship between exposure to wildfire smoke and long-term respiratory health consequences. An increasing body of research suggests that significant health impacts often occur with a delay, even if acute symptoms such as coughing, wheezing, and shortness of breath may appear within hours of exposure. This delayed reaction is significant for susceptible groups, such as people with cardiovascular diseases, asthma, and chronic obstructive pulmonary disease (COPD).

For instance, one study emphasized the importance of considering delayed effects in epidemiological models, noting that emergency department visits for respiratory disorders often peak one to two days after the most significant concentrations of PM_2.5_ from wildfire smoke [9]. Their comprehensive analysis emphasizes the methodological necessity of distributed lag models to account for the delayed and cumulative impact of exposure, particularly in susceptible groups, such as Medicaid participants. Symptoms, including respiratory discomfort, nasal passage irritation, and airway discomfort, can also worsen or persist longer after the apparent smoke has cleared [10]. Compared to normal urban pollution, wildfire particles cause greater inflammation, which may have long-lasting effects that patients and doctors may not notice immediately.

The EPA stated that brief encounters with wildfire smoke, lasting anywhere from a few hours through several days, might raise the possibility of bronchitis, exacerbate asthma, and impair lung function [11]. The organization also points out that hospital stays and emergency room visits are frequently postponed after smoking incidents involving a wildfire, which emphasizes the necessity of temporally sensitive surveillance and intervention techniques.

Public health preparation requires an understanding of these delayed consequences. Real-time air quality alerts may not capture the entire window of risk, potentially leaving healthcare systems underprepared and missing opportunities for early intervention. Public health organizations can accurately forecast spikes in hospitalizations for respiratory conditions and allocate resources more effectively by incorporating delayed exposure-response correlations into their predictive models. Targeted communication that encourages people to keep an eye on their symptoms for a period of time after exposure, even if the air quality improves, can also promote self-care and lessen delayed effects. This strategy is especially relevant for communities with limited access to healthcare or those residing in rural areas, such as Wyoming, where wildfire smoke may linger longer due to physical and climatic variables. 

This study builds on these findings by positing that in frontier environments, this lag is significantly extended. We hypothesize that structural barriers in Wyoming push the "Release" of healthcare demand beyond the standard 48-hour window observed in urban centers, necessitating our focus on the whole seven-day post-episode period.

Vulnerable Populations and Health Disparities

When it comes to health concerns associated with exposure to wildfire smoke, Medicaid users are a particularly vulnerable group within the total population. Medicaid is intended for low-income people, many of whom also suffer from exacerbated social as well as ecological challenges. These individuals face a greater risk of living in areas with unhealthy air quality or inadequate infrastructure to mitigate smoking exposure, poor housing conditions, and limited access to healthcare [12].

Many Medicaid recipients are members of populations who are inherently socially or biologically vulnerable to the negative health consequences of PM_2.5_, including children, the elderly, and those with long-term respiratory or cardiovascular disorders. Medicaid-eligible children, for instance, are more likely to reside in places with greater pollution levels and have higher asthma rates, both of which can be made worse by smoke from wildfires [13]. Due to difficulties with transportation or provider shortages, older persons on Medicaid may get treatment that is delayed or divided, which raises the risk of uncontrolled symptoms during smoke episodes.

The health effects of exposure to wildfire smoke are significantly influenced by socioeconomic level (SES) and healthcare accessibility. Medicaid beneficiaries are disproportionately impacted, as they frequently represent low-income and medically disadvantaged communities. According to studies, individuals in lower socioeconomic groups have less access to protective structures, healthcare services, and air filtration, resulting in higher exposure levels and a diminished ability to manage hazards [14]. For instance, people who work outside or live in subpar housing, which is common among Medicaid populations, are exposed to hazardous conditions for extended periods without proper protection, leading to increased emergency room visits and hospital stays [15].

Systemic racism and residential segregation are two fundamental variables that exacerbate environmental health inequities. Due to discriminatory zoning and land-use practices, low-income and communities of color are more likely to be located close to pollution sources and have fewer adaptive resources [16]. Cumulative exposure to multiple environmental risks these populations frequently endure increases the health burden during wildfire outbreaks. According to research, even Black people with higher incomes are at more risk than White people, indicating that structural injustices and ongoing stress are factors that increase susceptibility in addition to financial position [17].

Integrating equity-focused frameworks into wildfire preparation plans and public health initiatives is crucial for addressing these disparities. Medicaid populations and other underprivileged groups should be provided a focus in policy when it comes to protective measures, such as enhanced air quality monitoring, focused outreach, and access to medical and mental health treatment during smoke events. Research and policymakers should focus on the relationship between environmental exposure and social vulnerability as climate change increases the frequency and intensity of wildfires. 

These socioeconomic determinants serve as the primary "suppression" mechanisms in our theoretical framework. Factors such as a lack of dependable transportation or the inability to miss work do not just delay care; they effectively hide the true incidence of respiratory distress until the environmental crisis has subsided.

Medicaid's Role in Exposure-Response Research

Medicaid administrative claims data serve as a vital tool in exposure-response research by providing a high-resolution, longitudinal record of healthcare encounters for populations most susceptible to environmental stressors. Unlike aggregate public health surveillance, which may capture only catastrophic outcomes such as mortality or major hospitalizations, Medicaid claims allow researchers to track a broad spectrum of respiratory morbidity, including primary care visits, pharmacy fills, and outpatient management of chronic conditions. For the frontier population of Wyoming, these claims function as a critical proxy for health-seeking behavior in a demographic that often lacks "environmental buffers," such as home air filtration or the ability to socially distance from smoke hazards in climate-controlled environments.

By using this specific data source, researchers can bridge the "surveillance gap" in rural health. While traditional studies often focus on urban centers where healthcare access is immediate, Medicaid claims capture the specific healthcare utilization patterns of individuals navigating the structural inequalities of rural systems.

In the context of this study, Medicaid data are the essential empirical marker for testing the "Suppression and Release Cycle." Because Medicaid recipients are disproportionately affected by barriers such as transportation costs and the lack of paid leave, their claims records do not just reflect biological symptoms - they also reflect the timing of those symptoms as dictated by environmental and socioeconomic constraints. By analyzing these claims in the post-episode period, we can identify how the removal of ecological barriers (the "Release" phase) leads to a measurable surge in healthcare demand that would otherwise remain hidden in broader, less granular datasets.

Public Health Surveillance and Data Systems

The CDC’s National Environmental Public Health Tracking Network (NEPHTN) provides integrated data on environmental exposures and health outcomes, including air pollutants such as PM_2.5_ and related respiratory disorders [18]. Users of NEPHTN can investigate regional and time-varying patterns in air quality and health indicators, including emergency department visits and hospitalizations for asthma and COPD. Additionally, it includes real-time air quality surveillance and modeled wildfire smoke predictions, both of which are becoming increasingly important given the intensity and recurrence of wildfires.

Wyoming has a less centralized and constrained surveillance system. Wyoming's Department of Environmental Quality (WDEQ) monitors pollutants such as PM_2.5_, PM_10_, and O_3_ throughout the state. When wildfires occur, these publicly accessible data are utilized to issue health advisories, frequently in conjunction with the National Weather Service (NWS) and the Wyoming Department of Health (WDH). When PM_2.5_ concentrations exceed 25 micrograms per cubic meter (µg/m³) or when heavy smoke is predicted, an air quality alert is issued [19]. Advisories are directed towards vulnerable groups, including children, the elderly, and those with underlying respiratory disorders.

Nonetheless, Wyoming's capacity to monitor respiratory illnesses directly associated with wildfire smoke remains limited. Although WDH offers general health advice during smoke events, its health-related surveillance systems do not integrate with environmental exposure data. They are primarily intended for monitoring infectious and communicable diseases. There is no single platform that connects exposure to PM_2.5_ from wildfires to immediate respiratory flare-ups, emergency room visits, or Medicaid claims. The state's capacity to assess the acute health repercussions is hindered by this absence, particularly for vulnerable individuals in rural areas.

Furthermore, compared with other types of air pollution, wildfire smoke poses particular difficulties. Even in locations distant from the fire source, exposure risk is increased by the high concentrations of reactive gases and delicate particulate matter that can travel long distances and remain in the atmosphere for extended periods. One study found that wildfire smoke has reversed decades of progress in lowering ambient PM_2.5_ concentrations, increasing levels by 25-50% in nearly 75% of the U.S. [20]. Wildfire smoke has been shown to cause sudden increases in asthma inhaler sales and emergency room visits [20]. This highlights the need for responsive monitoring systems that can identify and connect these health events to environmental data.

Improved integration between health outcome data and air quality monitoring is essential in strengthening Wyoming's public health response. Real-time tracking and equity-focused interventions could be further developed by extending syndromic surveillance to encompass environmental triggers, integrating Medicaid claims analysis, and utilizing national platforms such as NEPHTN. Furthermore, more precise and rapid evaluations of the health effects of smoke would be possible if portable air sensors were placed in areas that are vulnerable to wildfires and connected to health data systems. 

This absence of integrated, real-time tracking reinforces the need for our study’s retrospective analysis of the post-episode surge. By identifying a predictable "release" phase in the claims data, this research provides the evidence needed to bridge the gap between Wyoming’s current air quality alerts and the delayed medical emergencies they fail to capture.

## Materials and methods

Study design

This study examined the acute public health effects of wildfire smoke exposure on Medicaid claims associated with respiratory conditions in Wyoming, using a longitudinal paired analysis. This design’s central component is a within-subject comparison, in which every incident of wildfire smoke acts as a separate control. This was accomplished by operationally defining a “wildfire smoke episode” as a duration of at least one day during which the county-level AQI was above 100 as a result of wildfire smoke.

The duration of the smoke episode (the 'during' period) and the seven days immediately following (the 'post7' period) were analyzed as distinct observation windows to capture the whole trajectory of the exposure-response relationship. A seven-day post-exposure window was selected based on established clinical evidence that the inflammatory response to PM_2.5​_ - including secondary complications such as bronchitis or pneumonia - often reaches clinical severity several days after the initial exposure. Furthermore, this window accounts for the 'Suppression and Release Cycle' hypothesized in this study: during active episodes, hazardous conditions and health advisories often suppress immediate healthcare-seeking behavior. Once environmental threats recede, the removal of these physical and psychological barriers triggers a concentrated 'release' or surge in delayed medical claims.

This paired-analysis methodology is a practical approach that enables direct comparisons of claims within the same wildfire event while efficiently controlling for episode-specific variables and patient-level factors that may otherwise distort results. A comparison of the number of claims in the ‘post7’ and ‘during’ periods for the same event allows us to evaluate the short-term shift in respiratory-related healthcare utilization. This method provides a comprehensive framework for assessing the immediate health impacts of wildfire smoke.

Data source: respiratory illnesses

This study examines the daily count of respiratory-related healthcare claims filed under the Wyoming Medicaid and Kid Care CHIP within the WDH. The selection process began by identifying a comprehensive yet particular subset of International Classification of Diseases, Ninth and Tenth Revision (ICD-9 and ICD-10) diagnosis codes (Appendix). This selection focused primarily on acute and chronic conditions known to be exacerbated by PM_2.5_​ exposure, including codes for asthma exacerbation, COPD, acute bronchitis, pneumonia, and other acute respiratory infections. 

The goal was to capture only utilization attributable to respiratory distress, excluding broad or nonspecific symptoms, as they lack the etiological specificity required for a robust environmental exposure analysis. To ensure data integrity, a secondary filtering process was applied: of the initial 4,022 identified smoke episodes, 1,224 were excluded due to incomplete AQI monitoring records or missing Medicaid claim identifiers. The final analysis was restricted to 2,798 high-confidence episodes where both environmental exposure and health utilization data were continuous across the 14-day study window (2012-2024).

For the mixed-effects modeling approach, the claim data needed to be aggregated from the patient level to the observation level. The primary methodological constraint inherent in using this secondary data was the inability to access individual-level patient records due to privacy limitations. Consequently, the data were aggregated into a daily count of claims per county, generating the total daily utilization within each county boundary. This aggregation serves two critical functions: first, it protects patient privacy; and second, it aligns the claims count with geographically aggregated air quality monitoring data, enabling the construction of county-specific wildfire episodes.

Data source: air quality

The environmental exposure variable for this study was derived from the U.S. EPA AQI database. Specifically, we extracted daily, county-level PM_2.5_​ measurements from June 1 to October 31 for each year from 2012 to 2024. These five months were deliberately selected because they capture the seasonal window for wildfire activity and associated smoke intrusions across Wyoming and the Intermountain West. PM_2.5​_ was chosen as the primary pollutant variable due to its well-established and scientifically irrefutable link to adverse cardiorespiratory health effects, owing to its small aerodynamic diameter (<2.5 micrometers), which allows deep penetration into the lung's alveoli and subsequent systemic absorption.

The raw PM_2.5_ concentration data were converted to the EPA's standardized AQI scale, providing a normalized, publicly accessible metric for air quality status. An AQI value of 100 or greater was used to define a wildfire smoke episode, representing air quality designated as "Unhealthy for Sensitive Groups" or worse. This threshold ensures that the analysis focuses on periods where the environmental determinant is severe enough to warrant public health action.

Data linkage

The primary component of our analysis was a meticulous data linking procedure that combined data from two different organizations: the EPA and the WDH, as well as Medicaid and CHIP. To achieve this, EPA's AQI data were first used to identify "wildfire smoke episodes" as the continuous time period in a particular county during which the daily AQI exceeded 100. These episodes were then linked to the claims data, which included service dates and the patients' counties of residence. We combined the total number of respiratory-related claims that transpired during the episode and the next seven days for each county and episode. Each row in the unified, episode-level dataset produced by this procedure reflected a distinct wildfire incident and its associated health consequences. This method enabled the precise correlation of claims data with environmental exposure data across time, providing a strong basis for our paired analysis.

Data preparation

A multi-step data preparation procedure was employed to organize the data for a paired analysis, which was conducted in R (version 4.3.2, R Foundation for Statistical Computing, Vienna, Austria). First, the readxl program was used to load the raw data from an Excel file (Microsoft, Redmond, WA, USA). The dataset was not suitable for a mixed-effects model, as it was initially in a "wide format" with distinct columns for the number of claims made during (Y_during) and after (Y_post7) each episode.

Thus, the tidyr package's pivot_longer() function was used to reshape the data into a "long format". This method created a new binary variable, time_period, indicating whether the claim count fell into the 'during' or 'post7' period, and combined the two claim columns into a single dependent variable, claims. Because it enabled the model to compare claim counts within each episode directly, this modification was crucial for a paired analysis strategy.

After reshaping, the categorical variables (county and month) in the dataset were verified to ensure they were appropriately structured as factors. Lastly, the continuous predictor variables duration_days, mean_aqi, and month were normalized to enhance model stability and facilitate interpretation. Each variable was scaled to have a mean of zero and a standard deviation of one during this process. In models with many predictors, this standardization phase is crucial because it helps prevent multicollinearity problems and ensures that each variable contributes equally to the model, thereby enhancing the efficiency of the optimization methods.

Statistical analysis

The selection of the final statistical framework followed a sequential diagnostic protocol to ensure the robust management of clustered count data. Initially, a linear mixed-effects model (LMM) and a Poisson mixed-effects model were evaluated to account for the nested structure of the data, utilizing a random intercept for episode_id to manage intra-episode correlation. Before final model selection, we formally tested for overdispersion - where the variance of the respiratory claims significantly exceeded the mean. Upon confirming that the data exhibited substantial overdispersion (α>0), which violates the assumptions of the standard Poisson distribution, the negative binomial generalized linear mixed-effects model (NB-GLMM) was adopted. The NB-GLMM explicitly addresses this by introducing an additional dispersion parameter, yielding more accurate confidence intervals and robust standard errors. By treating county and month as fixed effects within this framework, the model controls for seasonality and local factors while avoiding the standard-error underestimation inherent in traditional linear regression.

The purposeful incorporation of the NB-GLMM to directly address the crucial problem of overdispersion was a key feature of the study. Although the Poisson model is the industry standard for count data, its basic premise is that the variance equals the mean (variance = mean). This assumption is violated when there is overdispersion, which occurs when the variance of the data exceeds its mean. In the Poisson model, this would result in compressed standard errors and inflated test statistics (i.e., artificially tiny p-values). In contrast, the NB-GLMM accounts for this excess variance by adding an extra dispersion parameter (often denoted by θ or k), which enables the model to capture the underlying variability in the number of claims accurately. The NB-GLMM provides more accurate standard errors and more reliable conclusions about the impact of the time_period variable on respiratory claims, as it explicitly accounts for overdispersion.

The null hypothesis (H_0_) stated that there would be no statistically significant change in the number of claims if the coefficient for time_period were equal to zero. The idea that this coefficient is not equal to zero was the alternative hypothesis (H_1_). Coefficients, standard errors, and p-values were among the model outputs cleaned and saved to an Excel file using the broom, writexl, and mixed packages for the final report.

These models were fitted using R's lme4 and lmerTest (for LMM), and glmmTMB (for GLMMs). Model outputs, including coefficients, standard errors, and p-values, were cleaned up using the broom.mixed package and saved to an Excel file with the writexl package for the final report. All visualizations (including prediction plots for all three models) were created using the ggplot2 and ggeffects packages in R.

Visualizations

In conjunction with the statistical results, several visualizations were created to provide a comprehensive and precise representation of the data and model outcomes. Formally, the distribution of claims for respiratory conditions was described using a box plot, which provided a nonparametric summary of the median, quartiles, and range for each of the two observation periods. To show the raw, unadjusted link between the mean AQI and claims volume, an exploratory scatterplot was also created. A linear trend line was superimposed to show the early positive association. This stage was critical for making an initial assessment of the data's intrinsic structure. The final visualization was a model prediction plot based on the NB-GLMM. These graphics, which show the expected marginal mean number of claims for both the "during" and "post7" periods, effectively convey the model's main conclusion. After accounting for other model factors, the figure provides a convincing, statistically sound visual representation of the notable rise in claims observed in the seven days following a wildfire smoke event, with 95% confidence intervals.

## Results

Primary inferential analysis

The primary analysis, using an NB-GLMM, identified a profound temporal shift in healthcare utilization following exposure to wildfire smoke. The model revealed that the seven-day period immediately following a smoke episode ("post7") was associated with approximately five times higher claim rates (IRR = 5.02; 95% CI: [4.85, 5.19]; p < 0.001) than the active episode period. This indicates that, after controlling for duration and county-level factors, the peak burden on the healthcare system is a delayed, post-exposure phenomenon.

The model diagnostics confirmed a high degree of statistical reliability. The final dispersion parameter resulted in a ratio of 0.93, supporting the suitability of the negative binomial specification for overdispersed count data. By explicitly accounting for the non-negative integer nature of the claims data, this model successfully mitigated the overdispersion inherent in standard Poisson specifications.

Predictors of respiratory claims

In the adjusted NB-GLMM framework, episode duration emerged as the dominant predictor of respiratory claims (β = 0.20,p < 0.001), indicating that for every additional day of smoke exposure, the expected count of respiratory claims in the post-exposure window increases significantly. Conversely, mean AQI was not statistically significant after adjusting for other factors (β = 0.01, p = 0.098) (Table 1).

**Table 1:** Summary of Negative Binomial Generalized Linear Mixed-Effects Model (NB-GLMM) Results The findings of the NB-GLMM used to evaluate the count-based characteristics of the claims data are summarized in this table. Along with the county and month fixed effects, it displays the fixed effects, which include the main variables of interest (time_period, duration_days_scaled, and mean_aqi_scaled). It also reports an overdispersion ratio to assess the model's fitness.

effect	component	group	term	estimate	std.error	statistic	p.value	conf.low	conf.high	model_type
fixed	cond		(Intercept)	0.46	0.04	11.02	< 0.001	0.38	0.55	Negative Binomial Mixed-Effects Model
fixed	cond		time_periodpost7	1.61	0.02	93.83	< 0.001	1.58	1.65	Negative Binomial Mixed-Effects Model
fixed	cond		duration_days_scaled	0.2	0.01	29.11	< 0.001	0.18	0.21	Negative Binomial Mixed-Effects Model
fixed	cond		mean_aqi_scaled	0.01	0.01	1.71	0.098	0	0.03	Negative Binomial Mixed-Effects Model
fixed	cond		countyBig Horn	-0.78	0.21	-3.74	< 0.001	-1.19	-0.37	Negative Binomial Mixed-Effects Model
fixed	cond		countyCampbell	0.26	0.04	6.15	< 0.001	0.18	0.35	Negative Binomial Mixed-Effects Model
fixed	cond		countyCarbon	-1	0.25	-4.04	< 0.001	-1.48	-0.51	Negative Binomial Mixed-Effects Model
fixed	cond		countyConverse	-0.57	0.09	-6.15	< 0.001	-0.75	-0.39	Negative Binomial Mixed-Effects Model
fixed	cond		countyFremont	0.62	0.04	14.37	< 0.001	0.53	0.7	Negative Binomial Mixed-Effects Model
fixed	cond		countyGoshen	-0.17	0.08	-2.11	0.026	-0.33	-0.01	Negative Binomial Mixed-Effects Model
fixed	cond		countyJohnson	-1.26	0.18	-6.97	< 0.001	-1.62	-0.91	Negative Binomial Mixed-Effects Model
fixed	cond		countyLaramie	1.01	0.05	22.49	< 0.001	0.92	1.1	Negative Binomial Mixed-Effects Model
fixed	cond		countyLincoln	-0.5	0.12	-4.18	< 0.001	-0.73	-0.27	Negative Binomial Mixed-Effects Model
fixed	cond		countyNatrona	1.12	0.05	23.13	< 0.001	1.03	1.22	Negative Binomial Mixed-Effects Model
fixed	cond		countyPark	0.07	0.05	1.48	0.183	-0.02	0.17	Negative Binomial Mixed-Effects Model
fixed	cond		countySheridan	0.17	0.05	3.66	< 0.001	0.08	0.26	Negative Binomial Mixed-Effects Model
fixed	cond		countySublette	-1.62	0.06	-27.52	< 0.001	-1.73	-1.5	Negative Binomial Mixed-Effects Model
fixed	cond		countySweetwater	0.34	0.05	6.55	< 0.001	0.24	0.44	Negative Binomial Mixed-Effects Model
fixed	cond		countyTeton	-1.19	0.05	-21.97	< 0.001	-1.3	-1.09	Negative Binomial Mixed-Effects Model
fixed	cond		countyWashakie	-0.9	0.19	-4.69	< 0.001	-1.27	-0.52	Negative Binomial Mixed-Effects Model
fixed	cond		countyWeston	-0.79	0.23	-3.44	< 0.001	-1.24	-0.34	Negative Binomial Mixed-Effects Model
fixed	cond		month_scaled	0.05	0.01	7.09	< 0.001	0.04	0.07	Negative Binomial Mixed-Effects Model
ran_pars	cond	episode_id	sd__(Intercept)	< 0.001				10.27	12.58	Negative Binomial Mixed-Effects Model
diagnostic			Overdispersion_Ratio	0.93						

These findings suggest that the length of time a population is exposed to hazardous air and the subsequent duration of healthcare service suppression are more reliable predictors of the claims surge than the average intensity of the smoke alone. This implies a cumulative toxicological threshold: once air quality exceeds the "Unhealthy" benchmark, the biological and behavioral damage is driven more by the persistence of the insult than by incremental fluctuations in pollutant concentration. Consequently, long-duration, moderate-intensity smoke events may pose a greater threat to rural healthcare infrastructure than brief, high-intensity "spike" events that allow for faster recovery.

Descriptive statistics and contextual evidence

**Figure 1:**
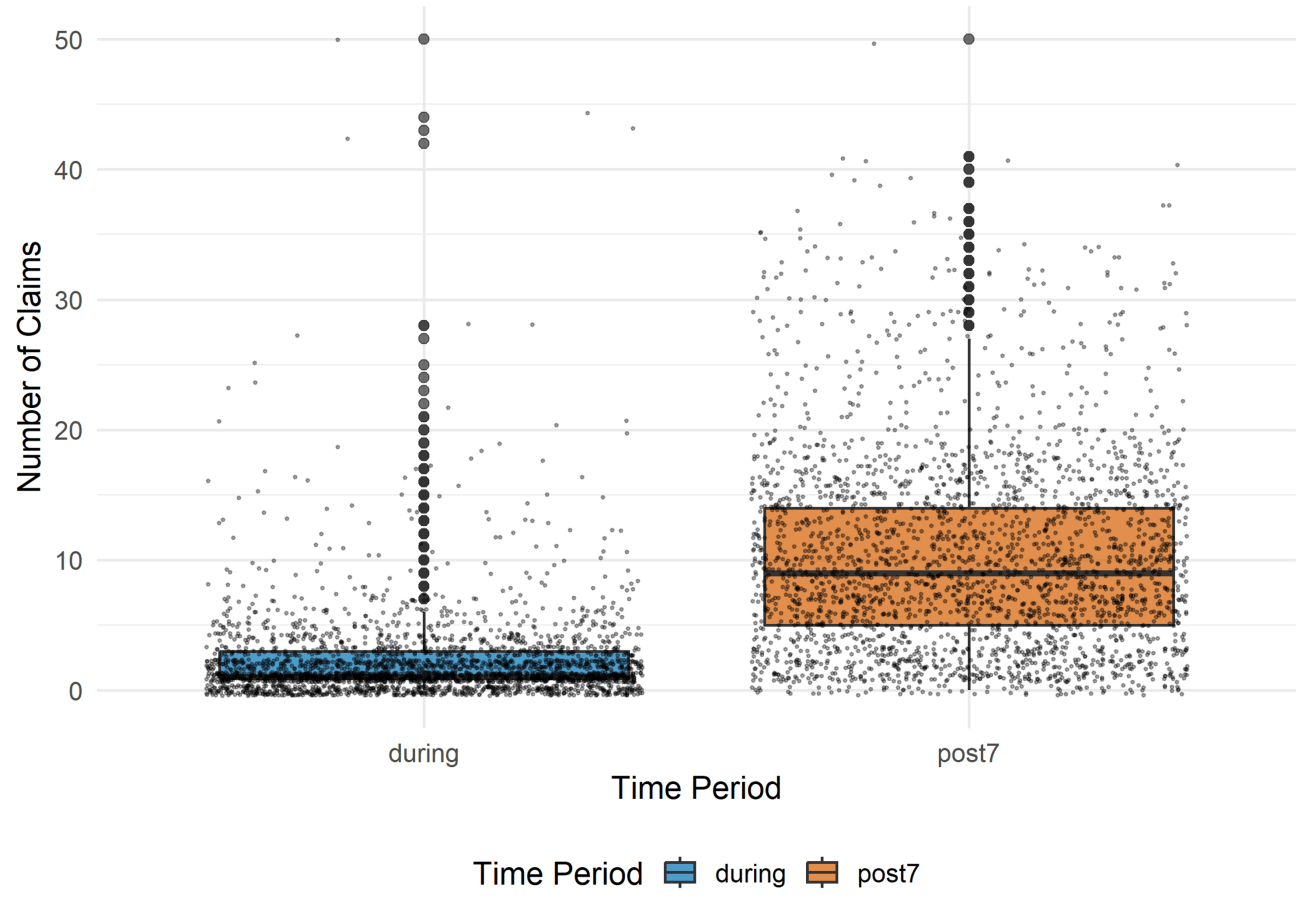
Distribution of Claims During vs. Post-Wildfire Episode This box plot compares Medicaid respiratory claims filed during the wildfire episode (“during”) and in the seven days following it (“post7”).

The descriptive data from 2,797 wildfire smoke episodes, totaling 5,594 paired observations, provide a robust empirical foundation for these inferential findings (Figure 1). During active smoke episodes, the mean daily respiratory claim count remained relatively suppressed, with a median density tightly clustered at the lower end of the utilization scale. In contrast, the subsequent "post7" window displayed a dramatically altered distribution, characterized by significantly broader variability and a higher median claim count.

This distributional shift suggests that while active smoke events exert a homogenizing effect on healthcare-seeking behavior - likely due to shared environmental barriers - the post-exposure period allows for a divergent "release" of demand. The increased variance in the post-exposure window further indicates that while most counties experience a surge, the magnitude of this rebound is modulated by local factors such as baseline population health and varying levels of rural healthcare access, resulting in the heavy-tailed distribution typical of overdispersed Medicaid data.

**Figure 2:**
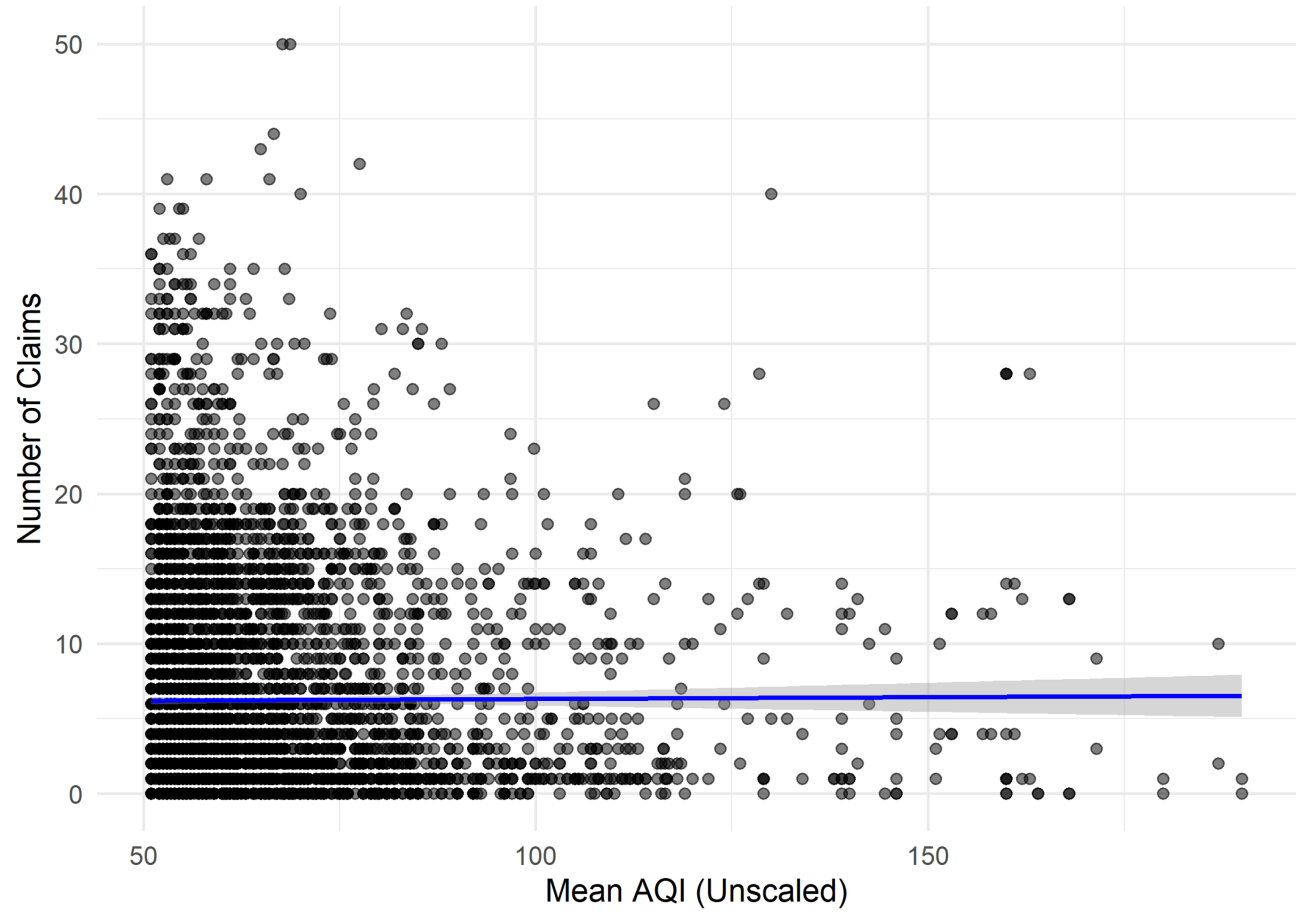
Claims vs. Mean Air Quality Index (AQI) During Wildfire Episodes This scatter plot shows the correlation between the mean AQI during wildfire smoke events and the number of Medicaid claims for respiratory conditions. Each point represents a single day during a wildfire incident, with the number of allegations plotted against the matching average AQI.

Contextually, a scatter plot of initial observations revealed a positive relationship between mean AQI and the aggregated quantity of claims, suggesting a general linear trend (Figure 2). While this establishes the foundational assumption that AQI-measured severity predicts public health effects, the considerable dispersion of data points around the regression line reinforces the model's finding that intensity alone is a relatively blunt instrument for prediction.

This high degree of non-constant variance - where the variance of claims increases at higher AQI levels - indicates that while smoke intensity sets the stage for a health crisis, it does not strictly dictate its timing or scale. Instead, the substantial "noise" in the scatter plot underscores the influence of latent variables, such as the Suppression and Release Cycle, which decouple the immediate environmental insult from the eventual healthcare utilization event. Consequently, these findings highlight the need to move beyond simple linear AQI-utilization models to incorporate more predictive temporal metrics, such as duration and the post-exposure lag.

Predicted claims and graphical certainty

**Figure 3:**
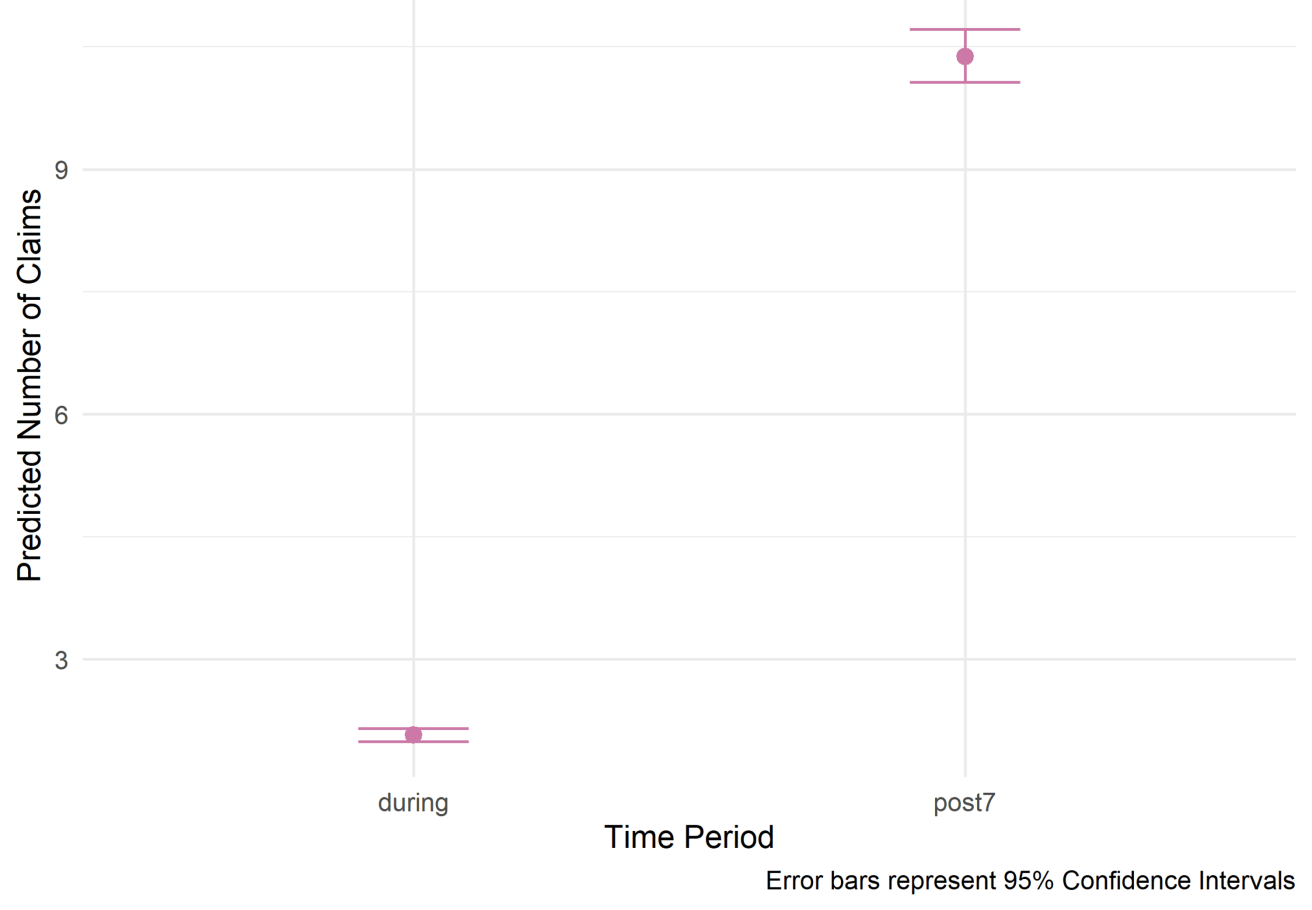
Predicted Claims From the Negative Binomial Model This plot displays the predicted number of claims for the "during wildfire episode" and "post-episode" periods based on the negative binomial generalized linear mixed-effects model (NB-GLMM). The height of each point represents the model's predicted mean number of claims, and the error bars indicate the 95% confidence interval.

The NB-GLMM estimates a stark escalation in magnitude: the predicted average during the wildfire episode is under two claims per day, while the expected average for the post-episode week surges to approximately eight claims (Figure 3). This fourfold increase in predicted daily volume highlights the profound impact of the "Release Phase" on local healthcare infrastructure.

By quantifying this shift, the model demonstrates that the most significant strain on respiratory health services occurs not when the skies are at their darkest, but in the immediate aftermath when the cumulative physiological insult manifests clinically. This drastic leap from a baseline-level demand to an eight-claim average represents a critical surge capacity challenge for frontier clinics, which are often staffed and resourced for the lower volumes observed during the active suppression phase.

From an inferential perspective, the 95% confidence intervals (CIs) for these periods are entirely disjoint, exhibiting a clear and significant separation in the parameter space. The lack of overlap between the "during" and "post7" CIs provides definitive graphical confirmation that the surge in healthcare demand is robust and predictable, independent of random variation or sampling error.

This statistical distinctness underscores the reliability of the observed phenomenon, confirming that the transition from the suppression phase to the release phase is not a subtle trend but a fundamental shift in the utilization profile. Because the lower bound of the post-exposure interval remains substantially higher than the upper bound of the active episode interval, these findings provide strong confidence to public health officials tasked with allocating emergency resources during the critical seven-day window following smoke dissipation.

## Discussion

The suppression and release cycle: a theoretical framework

The central contribution of this study is the identification of a "Suppression and Release Cycle" that defines the temporal relationship between wildfire smoke and healthcare utilization in frontier contexts. This framework moves beyond simple correlation to explain why respiratory-related Medicaid claim rates were approximately five times higher (IRR = 5.02) in the seven days following an episode than during the episode itself. According to the social-ecological model (SEM), the active phase of a smoke event induces a "Suppression Phase." During this time, high perceived barriers - such as hazardous driving conditions with low visibility and the economic risk of missing work - deter rural Medicaid beneficiaries from seeking immediate care. This behavioral suppression, bolstered by adaptive strategies such as sheltering in place, effectively masks the actual public health burden while physiological damage accumulates.

The "Release Phase" occurs immediately after the environmental threat recedes, marking a critical transition from hidden morbidity to visible healthcare demand. This surge is driven by two convergent forces: the conclusion of a biological latency period and the removal of structural impediments. As the visible smoke clears, the perceived barriers to transportation and clinic access - such as hazardous driving conditions and limited visibility - diminish just as the internal inflammatory damage triggered by PM_2.5_-induced oxidative stress and epithelial barrier disruption reaches a clinical threshold.

This delay is further compounded by the "weathering" effect on the respiratory system, where the cumulative toxicological load from the preceding days finally overpowers the body’s compensatory mechanisms. The result is a concentrated influx of patients whose conditions, having deteriorated and progressed during the suppression phase, often require more resource-intensive intervention - such as emergency nebulizer treatments or corticosteroids - than if treatment had been accessible during the initial exposure. Consequently, the release phase represents not just a spike in volume, but a spike in the clinical acuity of the cases being presented to the healthcare system.

Predictors of utilization and model suitability

Beyond the primary temporal shift, the study highlights the complexity of exposure metrics. Episode duration emerged as the dominant predictor of claims (p < 0.001), aligning with the global literature, which suggests that cumulative exposure is the primary driver of airway inflammation and bronchial irritation. Interestingly, while descriptive data showed a positive correlation between AQI and claims, mean AQI failed to reach statistical significance in the final adjusted NB-GLMM (p = 0.098). This suggests that once a high-hazard threshold is met, the duration of the incident and the subsequent "release" period are more reliable predictors of healthcare demand than incremental changes in pollutant intensity. Furthermore, the model’s diagnostics supported the suitability of the negative binomial specification for these overdispersed count data, ensuring that the reported standard errors and confidence intervals accurately reflect the rural Wyoming context.

Prioritized policy recommendations and economic impact

The 5.02-fold increase in post-exposure claims necessitates a fundamental shift in emergency preparedness. These findings suggest that the WDH and local clinics should prioritize interventions based on their immediate impact and feasibility.

Targeted Outreach (High Feasibility/Low Cost)

Public health advisories must not cease when the smoke clears. Targeted "Post-occurrence Outreach" via SMS and Medicaid provider networks should be sustained for seven days post-event to monitor vulnerable groups for delayed symptoms, such as persistent wheezing or sleep disruption.

Healthcare System Preparation (Moderate Feasibility)

Clinics should adjust emergency response windows to mobilize staffing and stock essential respiratory medications (e.g., bronchodilators and corticosteroids) specifically for the week following an incident.

Structural Mitigation (Long-Term Impact)

To prevent the "suppression" of care, the state should invest in HEPA filtration subsidies for Medicaid households and establish "Clean Air Hubs."

Given that a single respiratory-related emergency visit can cost significantly more than the price of a HEPA filter or an automated SMS alert system, shifting resources toward the post-episode period represents a powerful public health investment with the potential to yield substantial cost savings.

Limitations and future directions

Despite the robust statistical findings, several limitations must be acknowledged. Primarily, this study is subject to the ecological fallacy; because county-level AQI was used as a proxy for exposure, these community-level metrics may not reflect the actual individual-level dose inhaled by residents, particularly in geographically vast rural counties where a single monitoring station may be miles from a population center.

Furthermore, the model could not account for unobserved residual confounding, such as indoor air quality, individual-level protective behaviors (e.g., masking or air filtration usage), or the baseline severity of pre-existing comorbidities. We also could not fully control for interannual fluctuations in concurrent viral outbreaks, such as influenza or respiratory syncytial virus, which may independently drive respiratory claims during wildfire season.

Crucially, this analysis is subject to temporal ambiguity regarding the "date of service" recorded in Medicaid claims. While we observe a surge in the post-exposure window, these dates may reflect administrative scheduling delays - such as the time required to secure an appointment or transportation in rural areas - rather than a purely biological lag in symptom onset. Consequently, the observed 5.02 IRR may represent a combination of delayed physiological response and structural impediments to immediate care.

Future research should move beyond aggregate metrics by integrating individual-level data, linking anonymized patient addresses with high-resolution satellite-derived PM_2.5_ estimates and chemical transport models. This granular approach would address current spatial limitations and enable a more precise dose-response analysis that accounts for intra-county variability in smoke plume density. Furthermore, conducting a formal economic cost analysis of the reported eight-claim-per-day surge in the post-episode period is essential. By quantifying both the direct expenditures associated with delayed acute care and the indirect societal costs - such as lost labor productivity and caregiver burden - researchers can provide policymakers with a clear fiscal mandate to invest in prevention-based wildfire response strategies. Finally, prospective studies should evaluate the efficacy of specific interventions, such as distributing HEPA filters or targeted telehealth triage, to determine which multi-level policies most effectively flatten the post-exposure utilization curve.

## Conclusions

This research characterizes the respiratory health burden of acute wildfire smoke on the Medicaid-enrolled population, demonstrating a significant and predictable association between smoke exposure and increased healthcare utilization. The central contribution of this study is the identification of a pronounced "Suppression and Release" cycle. Rather than a steady increase in utilization, the data reveal a stark temporal shift: respiratory-related claims do not peak during the smoke event but escalate substantially in the following week. The final negative binomial mixed-effects model quantifies this impact, showing that claim rates are approximately five times higher (IRR = 5.02; 95% CI: [4.85, 5.19]; p < 0.001) in the seven-day period following a smoke event compared to the period of active exposure.

To address the profound vulnerabilities revealed by this lagged effect, the WDH should transition from reactive to proactive intervention strategies. Specifically, the department should implement an automated "Post-Exposure Outreach" protocol that triggers targeted SMS health alerts and clinical follow-up prompts to Medicaid beneficiaries for seven days after a county-level AQI alert ends. Additionally, the state should prioritize distributing subsidized HEPA air filtration units to high-risk Medicaid households, specifically in counties with significant fixed effects, such as Natrona and Laramie, to mitigate the physiological "dose" during the suppression phase and reduce the subsequent burden on the state's healthcare infrastructure.

## Appendices

**Table 2:** International Classification of Diseases (ICD)-10-CM and ICD-9-CM Clinical Coding Classifications for Respiratory-Related Medicaid Claims

International Classification of Diseases				Clinical Category
ICD-10		J40		Bronchitis, not specified as acute or chronic
		J43		Emphysema
		J44		Other chronic obstructive pulmonary disease
		J45		Asthma
		J47		Bronchiectasis
		J80		Acute respiratory distress syndrome
		R06.03		Acute respiratory distress
		P22.0		Respiratory distress syndrome of newborn
		X01.1XXA		Exposure to smoke in uncontrolled fire, not in building or structure, initial encounter
ICD-9		490		Bronchitis, not specified as acute or chronic
		491		Chronic Bronchitis
		492		Emphysema
		496		Chronic airway obstruction, not elsewhere classified
		493		Extrinsic asthma, unspecified
		493.1		Intrinsic asthma (nonallergic asthma)
		493.2		Chronic obstructive asthma
		493.5		Unspecified Asthma
		493.6		Asthma, induced by unspecified agent
		493.7		Unspecified asthma, unspecified
		493.8		Other specified asthma
		493.9		Asthma, unspecified
		494		Bronchiectasis without acute exacerbation
		494.1		Bronchiectasis with acute exacerbation
		494.8		Other specified bronchiectasis
		518.82		Other pulmonary insufficiency, not elsewhere classified
		799.1		Respiratory arrest
		E890		Conflagration (uncontrolled fire) in a private dwelling
